# Acute technostress, schizotypal traits, and visual illusion perception in the general population

**DOI:** 10.1007/s00221-026-07246-5

**Published:** 2026-02-24

**Authors:** Tri Nghia Le, Philippe A. Chouinard, Adam Abou-Sinna, Irene Sperandio, Katy L. Unwin

**Affiliations:** 1 Department of Psychology, Counselling, and Therapy, La Trobe University, Bundoora, Australia; 2 Department of Psychological Sciences, Swinburne University of Technology, Melbourne, Australia; 3 Department of Psychology and Cognitive Science, University of Trento, Trento, Italy

**Keywords:** Computer glitches, Technology-inducing stress, Schizotypy, Cognitive-perceptual schizotypy, Interpersonal schizotypy, Ponzo illusion, Ebbinghaus illusion, Müller-Lyer illusion, Poggendorff illusion, Bayesian predictive coding

## Abstract

The present study investigated the impact of acute technostress (i.e., stress induced by technology – here, computer glitches) and schizotypy on visual illusions, including the Müller-Lyer, Ebbinghaus, Poggendorff, and Ponzo illusions. While previous research has examined perceptual anomalies in schizophrenia and schizotypy, the role of stress in perceptual distortions has been overlooked until recently. In particularly, its acute effects are unknown. Healthy participants completed four visual illusion tasks under two conditions: a standard condition and a technostress-inducing condition involving unpredictable computer glitches. The Galvanic Skin Response (GSR) was recorded as a physiological measure of stress and levels of schizotypy were assessed using the Schizotypal Personality Questionnaire (SPQ). The interpersonal schizotypal subscale was negatively associated with the strength of the Ponzo and Ebbinghaus illusions in both stress conditions while the strength of Poggendorff illusion decreased in both stress conditions with the cognitive-perceptual schizotypy subscale. In addition, a positive relationship emerged between the Müller-Lyer illusion and GSR under the technostress condition. This relationship was not present in the condition without technostress. Conversely, there was a trend for the Poggendorff illusion to increase with GSR in the condition without technostress. This relationship was not present in the condition with technostress. These findings reveal how acute technostress and different components of schizotypy can exert different effects on different illusions, presumably because different mechanisms underlie the different illusions. Our study highlights the relevance of everyday technological stressors for visual perception and underscore the need for future studies to clarify further how stress, schizotypal traits, and perceptual inference are linked mechanistically.

## Introduction

Schizophrenia is a psychiatric disorder characterised by negative symptoms (e.g., decreased feeling of pleasure – “anhedonia”), disorganised behaviour and speech, and experiences of delusions or hallucinations (American Psychiatric Association 2013). Although perceptual functions are frequently impaired in schizophrenia, there has been less work to understand these deficits in the visual domain (Butler et al. 2008). One way to conceptualise schizophrenia and its related perceptual deficits is through a dimensional schizophrenia spectrum of schizotypal traits, or schizotypy (van Os et al. 2009). Under this framework, schizophrenia exists at the most severe clinical end of the spectrum. Meanwhile, at the non-clinical end of the spectrum, schizotypy represents a set of schizotypal-personality traits reflecting the milder and subclinical expression of schizophrenia in the general population (e.g., odd or bizarre behaviour, unusual perceptual experiences, and ideas of reference; Claridge 1997). Accumulating evidence supports the overlap in genetic, neural, and behavioural factors between schizotypy and schizophrenia (Nelson et al. 2013). Consequently, researchers have used schizotypy as a model to investigate the complexity of schizophrenia pathogenesis. Past literature highlights how visual-perceptual abnormalities in schizophrenia manifest at a milder level within the spectrum of schizotypy (Ettinger et al. 2014, 2015). Thus, targeting schizotypy in research of visual perception is a useful approach for garnering a more comprehensive understanding of how visual-perceptual deficits might develop and progress along the schizophrenia spectrum.

Perceptual organisation, which involves processes by which visual components are combined into coherent object representations, is one of the perceptual deficits found along the schizophrenia spectrum (Butler et al. 2008). Visual illusions are a useful tool for understanding deficits in perceptual organisation in the schizophrenia spectrum because they tap into visual binding mechanisms that involve the interaction between bottom-up and top-down processing (for reviews, see Notredame et al. 2014; King et al. 2017). More precisely, visual illusions depict a mismatch between sensory input and perceptual experiences (Gregory 1997). This mismatch arises from a misinterpretation of bottom-up sensory information due to top-down contextual influences, causing perception to diverge from physical reality (Gregory 1997). Following this reasoning, a change in this interaction would result in a change in illusion strength – making visual illusions a useful tool for examining how perceptual processing changes with a condition or in people with particular traits associated with the condition (Landry and Chouinard 2016).

Earlier research on the relationship between schizotypy and visual illusions is limited but seems to align with what is demonstrated in schizophrenia. Bressan and Kramer (2013) found reduced illusion strength in the Ebbinghaus illusion in individuals with high positive schizotypy. However, this study focused solely on the positive dimension of schizotypy, without accounting for other domains of schizotypy, such as negative schizotypy or disorganized traits. This reduction in illusion strength aligns with many studies in clinical populations across the psychosis spectrum. Most literature on visual illusions suggests that individuals with schizophrenia are less susceptible to illusions and perceive them closer to their physical reality (King et al. 2017). This aligns with the hypothesis that schizophrenia and high schizotypy is linked to a weakening in top-down processing, which would increase a resistance to illusory effects (Hemsley 2005; Dima et al. 2010).

However, these findings do not always emerge. Some studies have demonstrated an increase rather than a decrease in illusion strength in individuals living with psychotic conditions (e.g., Sperandio et al. 2023; Capozzoli and Marsh 1994; Diržius et al., 2013; Chen et al. 2011). To explain these inconsistencies, symptom-specific research has highlighted how negative symptoms (e.g., avolition, anhedonia) can reduce illusion strength, which is consistent with a diminished reliance on priors and a greater fidelity on sensory information (Tschacher et al. 2006), whilst positive symptoms (e.g., hallucinations, delusions) can increase the strength of illusions, suggesting an overweighting of priors (Tschacher et al. 2006; Yang et al. 2013). This duality illustrates that perceptual alterations in psychosis cannot always be captured by a “weaker priors” account. Rather, in some instances, perceptual distortions can arise from a stronger influence of priors on visual perception.

The psychosis Bayesian predictive coding model provides an explanation for increases in illusion strength with positive symptoms (Fletcher and Frith 2009). This model posits that individuals with schizophrenia weigh prior beliefs stronger than true sensory evidence, leading to a heightened bias toward beliefs and consequently, hallucinatory or delusional experiences (Sterzer et al. 2018). Rather than a weakening of top-down processing, individuals with schizophrenia-spectrum conditions exhibit an excessive reliance on prior beliefs, potentially explaining both positive symptoms and increased susceptibility to visual illusions, as sometimes reported in the literature (e.g., Sperandio et al. 2023; Capozzoli and Marsh 1994; Diržius et al., 2013; Chen et al. 2011).

Recent studies suggest that stress could be another factor to account for contradictions between studies demonstrating a decrease in illusion strength and those that demonstrate an increase instead. While much research has focused on stress as a risk factor for the onset of psychosis (Zubin and Spring 1977), we are unaware of any studies that have examined its immediate perceptual consequences. More recently, it has been suggested that stress itself may reflect the effects of acute, momentary positive symptoms in young people at ultra-high risk for psychosis (Cavelti et al. 2025). Stress is often operationalised as the psychological or physiological response to a real or perceived stressor that threatens homeostasis (Schneiderman, 2005). In terms of chronic stress, Sperandio et al. (2023) examined a set of visual illusions in young adults with psychotic-like experiences and found that they exhibited greater susceptibility to the illusions compared to a control group. Notably, the study demonstrated that it was the participants’ stress levels, rather than their psychotic-like traits, that best predicted increases in illusion strength. Similarly, Zouraraki et al. (2023) revealed a positive association between the Müller-Lyer illusion and schizotypal traits, but only in individuals who experienced high levels of trauma – an extreme form of stress. These findings challenge claims that individuals on the schizophrenia spectrum have weaker top-down processing. Rather, they suggest that chronic stress may induce opposite effects.

The Overarching States of Mind framework provides an explanation for increases in illusion strength with stress (Herz et al. 2020). The model posits that stress narrows perception, hindering individuals from exploring alternative cues, and consequently, leading them to act upon pre-planned actions. It has also been proposed that an increase in cognitive load from stress can direct people to be misguided by prior beliefs (Baror and Bar 2022). Based on these theoretical perspectives, it is possible that stress and schizotypal traits may jointly increase susceptibility to visual illusions in the general population.

In the modern world of digitalisation, stress can commonly manifest through technology use in the form of technostress. Technostress refers to a prominent psychological stress caused by technology-related hassles (Tacy 2016). Past studies demonstrate that acute technostressors, such as system breakdowns or computer glitches, can lead to changes in arousal, as measured by increases in skin conductance (Mishra and Rašticová 2024). With technostress becoming more common, especially after COVID-19 with the increased use of technology, studying its acute effects on visual perception can help understand its impact on the everyday visual experiences of people with schizotypy.

**Fig. 1:**
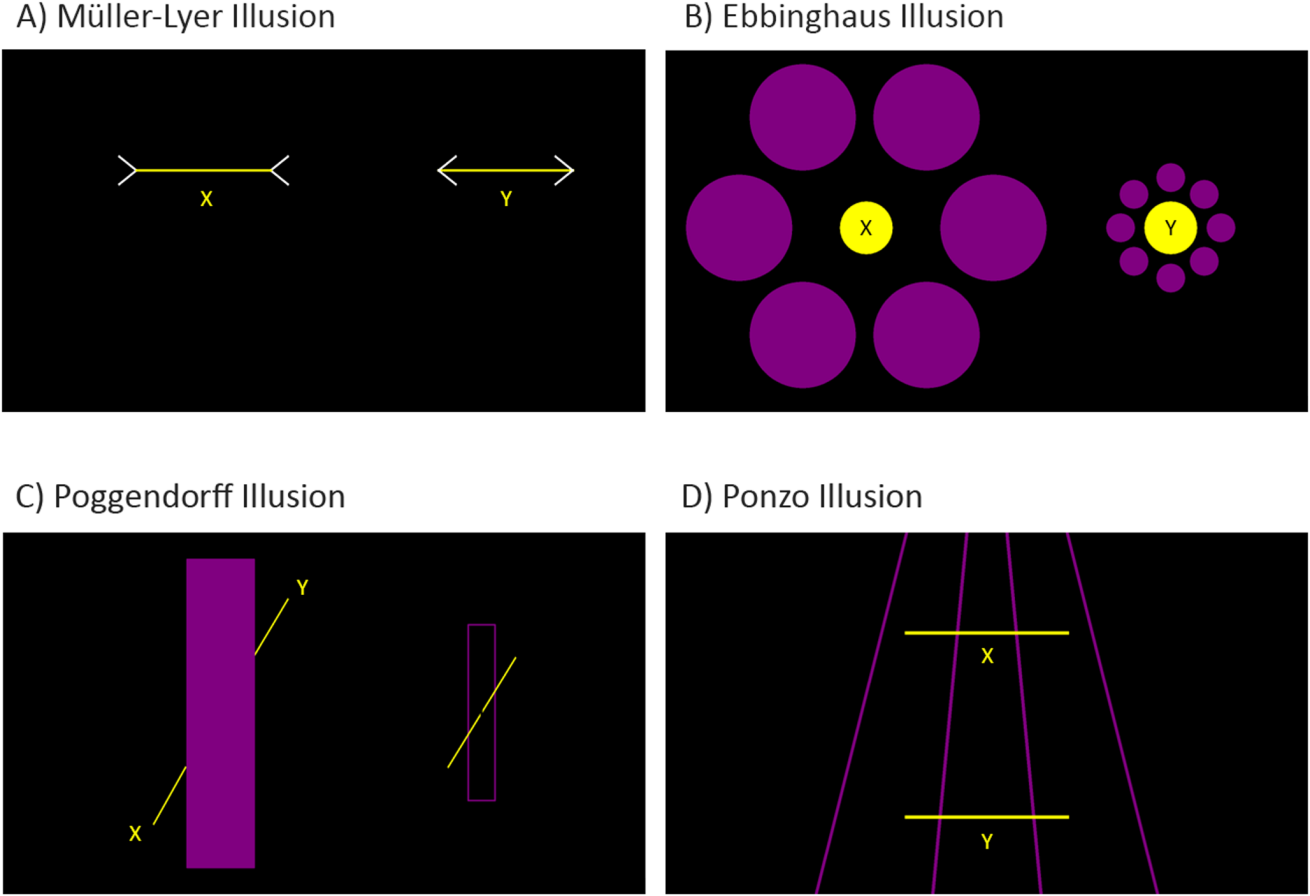
The visual illusions. We examined four illusions: the Müller-Lyer (**A**), Ebbinghaus (**B**), Poggendorff (**C**), and Ponzo (**D**) illusions. The labels X and Y were added to this figure to illustratively denote the stimuli that were designated as either the comparison or standard stimuli. For each trial, participants had to adjust the comparison stimulus to appear the same size as the standard stimulus (A, B, and D) or continuing along the same collinear path as the standard stimulus (**C**). The examples in this figure illustrate how the illusion displays appeared to participants when the comparison and stimuli had the same size (**A**, **B**, and **D**) or were collinear to each other (**C**). Note that a template picture (on the right of the display) was provided for the Poggendorff illusion to facilitate participant’s understanding of the task

Despite the wealth of research on visual illusions, it remains unclear how stress, particularly acute stress, and schizotypal traits influence their perception. In this study, we selected four visual illusions that have been studied extensively in patients with schizophrenia: the Müller-Lyer, Ebbinghaus, Poggendorff, and Ponzo illusions (Costa et al. 2023; King et al. 2017; Notredame et al. 2014) (Fig. 1). Research on visual illusions in schizophrenia has often treated, albeit implicitly, visual illusions as arising from a shared underlying mechanism. However, one should not make this assumption. Our earlier research demonstrates how a principal component analysis on the illusion strength of 17 illusions gives rise to five separate components – indicating that some illusions tap into different mechanisms than others (Chouinard et al. 2016). Although the precise mechanism underlying each illusion remains contentious, there is a tendency for certain explanations to be more prominent than others. For instance, size-contrast explanations (Gold 2014) seem more prominent in accounting for the Ebbinghaus illusion (Fig. 1B) (Sherman and Chouinard 2016), whilst perspective explanations (Gregory 1963) seem more prominent in accounting for the Ponzo illusion (Fig. 1D) (Yildiz et al. 2022). Based on our reading of the literature, explanations for the Müller-Lyer illusion (Fig. 1A) are particularly contentious but tend to be divided into non-perspective (Morgan et al. 1990) and perspective-based (Gregory 1963) accounts. The Poggendorff illusion (Fig. 1C) differs from the others in that the illusion pertains to a perceptual misalignment between lines rather than a perceptual rescaling of size, which conceivably could involve different mechanisms. Chouinard et al. (2021) have provided evidence that the Poggendorff illusion is influenced by both low and high levels of cognitive processing^1^.

In short, the link between acute stress, schizotypal traits, and susceptibility to visual illusions is largely underexplored. This study aims to investigate the influence of acute technostress and schizotypal traits on the strength of visual illusions in a sample from the general population. We hypothesise that susceptibility to visual illusions will be influenced by both technostress and schizotypal traits, with the strength of these effects varying across different illusions, which are underpinned by different mechanisms.

## Method

### Participants

Thirty-eight participants were recruited via convenience sampling through various methods including word of mouth, social media, and recruitment posters distributed throughout La Trobe University’s Bundoora Campus. All interested participants, who were mostly university students and academic staff, met the inclusion criteria. However, data from three participants could not be included due to recording issues. The final sample consisted of 35 participants, predominantly female (*n =* 25, 69.4%, *n* = 10 males), aged 18 to 46 years (*M* = 23.7, *SD* = 5.4). To be eligible for the study, participants had to be 18–60 years of age and right-handed. Participants were also required to have normal or corrected-to-normal vision, be free from migraines, and abstain from psychotropic or drowsiness-inducing medication. Only one participant declared having a diagnosis of autism. No record of other psychiatric or neurological conditions was found in our sample. Ethics approval was granted for this study by the La Trobe University Human Ethics Committee. Prior to participation, written informed consent was obtained from all participants.

### Overview of procedures

Participants underwent visual screening tests before performing a series of computerised tasks. The computerised tasks comprised the following in the following order: a handedness questionnaire, the visual illusion tasks, a demographics and health questionnaire, and a schizotypy questionnaire. These latter questionnaires were administered last to mitigate potential demand characteristics that could influence participants’ perceptual judgements. A desktop PC (Precision T1700, Dell Inc., Austin, TX, United States) running Windows 10 administered these tasks using E-prime 3 software (Psychology Software Tools, Sharpsburg, PA, USA) on a 21.5” liquid crystal display computer screen (Dell S2240T) with a resolution of 1920 × 1080 pixels, a pixel pitch of 0.248 × 0.248 mm, and a frame rate of 60 Hz. Participants were instructed to keep their head steady on a chin rest during task performance so that a viewing distance of 50 cm was maintained. Participants completed four visual illusion tasks under two stress conditions, one with computer glitches and the other without computer glitches. The order of these stress conditions was counterbalanced across participants. Each stress condition had four trials for each illusion. The order of trials within each stress condition was randomly generated in each participant. We measured the Galvanic skin response (GSR) throughout most of the testing session. The testing session took about 90 minutes. Participants were compensated for their time with gift vouchers.

### Visual screening tests

Participants were asked to read a Snellen chart from six feet away to assess visual acuity (Peters 1961). A minimum score of 20/40 was required to participate in the study. The Fly’s Stereopsis Test was also conducted to evaluate stereoscopic depth perception (De La Cruz et al. 2016). A minimum threshold of 40 s of arc was required to be eligible for the study.

### Questionnaires

To verify right handedness, the Flinders Handedness Survey (FLANDERS) was used (Nicholls et al. 2013). The questionnaire contains ten items to indicate whether participants use their left, right, or either hand to complete tasks (e.g., *“With which hand do you write?”*). Participants answered from three options: Left (-1), Right (+ 1), or Either (0). The item scores were summed to indicate their handedness: Lefthanded (-10 to -5), Righthanded (+ 5 to + 10), or Ambidextrous (-4 to + 4). Participants scoring at least + 5 were eligible. The FLANDERS has high internal reliability (α = 0.91). To characterise the sample, an in-house demographic questionnaire was created to collect information on participants’ gender identity, sex at birth, age, psychological and/or neurological diagnosis, and current medication use.

To evaluate schizotypy levels, the SPQ was used (Raine 1991). The SPQ is a 74-item questionnaire measuring dimensional schizotypy in the general population. It contains nine subscales based on the nine criteria for schizotypal personality disorder as defined in the Diagnostic and Statistical Manual of Mental Disorders, Third Edition, Revision (DSM-III-R). These subscales are Ideas of Reference, Social Anxiety, Odd Beliefs/Magical Thinking, Unusual Perceptual Experiences, Eccentric/Odd Behaviour and Appearance, No Close Friends, Odd Speech, Constricted Affect, and Suspiciousness/Paranoid Ideation. Participants answered “Yes” or “No” to each item based on their own experiences (e.g., “*Have you had experiences with the supernatural?*”). The nine subscales can be further mapped into three main factors: Cognitive-Perceptual Deficits, Interpersonal Deficits, and Disorganisation (Raine et al. 1994). All items endorsed with “Yes” were scored 1 point. The item scores were summed to create the total SPQ scores and three sub-scale scores for main factors, with higher scores indicating higher schizotypy. The Suspiciousness/Paranoid Ideation subscale was allocated exclusively to the Cognitive-Perceptual factor in this study, consistent with revised SPQ versions (e.g., SPQ-BR). In our sample, the SPQ demonstrated high internal validity ($\:\alpha\:=0.91)$. All three main factors of the SPQ showed high internal validity (Cognitive-Perceptual: $\:\alpha\:=0.88$; Interpersonal Deficits: $\:\alpha\:=0.88$; Disorganisation: $\:\alpha\:=0.86$).

### Procedures for all visual illusions

**Fig. 2:**
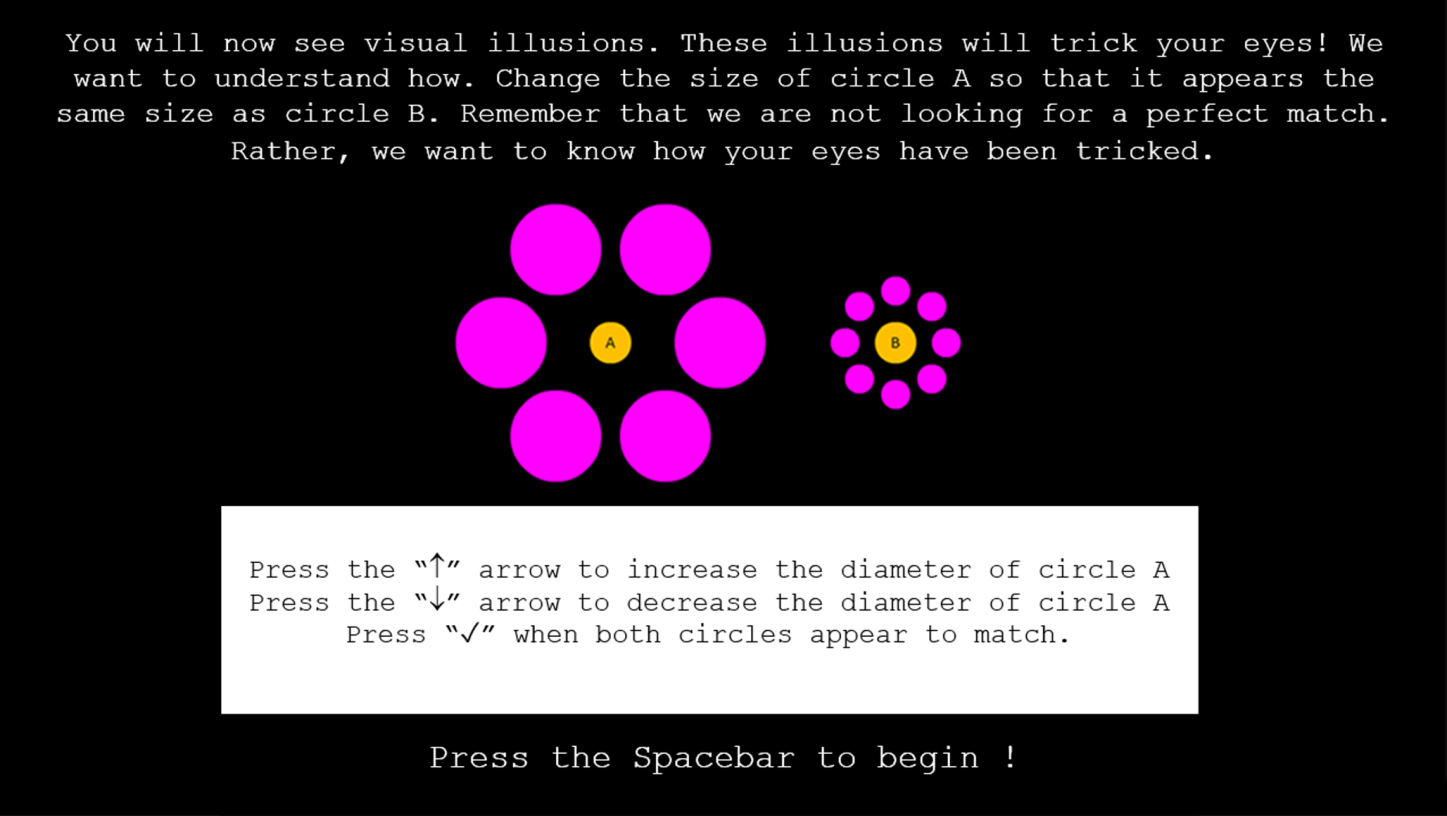
The instructions for the visual illusion task. Each trial began with instructions, such as in this example, informing participants which stimuli were designated as the comparison and standard, and how to adjust them with key pressing. The text and the illusion display were resized for this figure (i.e., the text was enlarged and the illusion display was shrunk to improve readability)

We examined susceptibility to four visual illusions: the Müller-Leyer, the Ebbinghaus, the Poggendorff, and the Ponzo illusions. Each illusion is shown in Fig. 1A-D. All illusions were presented over a black background (RGB: 0, 0. 0), with the displays maximised to full screen, subtending a visual angle of 54.6° in width and 30.7° in height. For each trial, participants adjusted a comparison stimulus to appear the same size or along a particular physical dimension as a standard stimulus by pressing keys labelled with up and down arrows – specifically, the ‘8’ and ‘2’ buttons on the numerical keypad marked with up and down arrow stickers. When participants were satisfied with their match, they pressed the ‘5’ button on the numerical keypad which was marked with a tick (✓) sticker. Participants were given as much time as they needed to complete each trial. Their final adjustment was recorded in pixels. Specific instructions were provided at the start of each trial, indicating which stimuli served as the comparison and standard stimuli. An example of an instruction is provided in Fig. 2. The instructions followed this general format:

*You will now see visual illusions. These illusions will trick your eyes! We want to understand how. Change the size of {COMPARISON DENOTED EITHER BY A OR B} so that it appears the same size as {STANDARD DENOTED EITHER BY B OR A}. Remember that we are not looking for a perfect match. Rather*,* we want to know how your eyes have been tricked.*

Computer glitches were used to induce technostress in the glitch condition. When participants performed the visual illusion task in the glitch condition, each keyboard press had a 1-in-6 chance of causing a two-second delay in responsiveness. The glitches were designed to be uncontrollable and unpredictable to efficiently induce acute stress (Dickerson and Kemeny 2004). They were intended to closely mimic daily hassles and effectively elicit technostress in a low-risk manner. In the non-glitch condition, no computer glitches were present.

### Procedures specific to the Müller-Lyer illusion

The display comprised two horizontal yellow lines (RGB: 255, 0, 255) with white arrowheads (RGB: 255, 255, 255) at both ends (Fig. 1A). The two configurations were presented side by side. The angle of the white lines comprising the arrowheads was 45° from the horizontal plane and their length subtended 0.2°. The arrowheads for the configuration on the left was always pointing inwards, while the arrowheads for the configuration on the right was always pointing outwards. The display typically produced the illusion that the yellow line on the left appeared longer than the one on the right when both were of the same length (Fig. 1A). One of the yellow lines was designated as the comparison, and the other as the standard. The standard line always subtended 11.4°, while the comparison line was initially presented at 1.2° or 23.9° when displayed on the left, and 5.7° or 17.0° when displayed on the right. The starting length of the comparison line and the assignment of stimuli as comparison or standard were randomised for each participant. The down and up arrow keys decreased and increased, respectively, the length of the comparison line by 0.06°. Final adjustments were recorded in pixels. These values represented the length that the comparison line needed to be to perceptually match the standard line.

### Procedures specific to the Ebbinghaus illusion

The illusion comprised two yellow circles (RGB: 255, 0, 255), each surrounded by contextual rings of purple circles (RGB: 128, 0, 128) (Fig. 1B). The two configurations were presented side by side. The configuration on the left always consisted of a ring of six contextual purple circles subtending 9.1° and the configuration on the right always consisted of a ring of eight contextual purple circles subtending 2.5°. The display typically produced the illusion that the yellow circle on the left appeared smaller than the one on the right when both were of the same diameter (Fig. 1B). One of the yellow circles was designated as the comparison, and the other as the standard. The standard always subtended 4.5°, while the comparison was initially presented with a diameter of 0.6° or 11.4° when displayed on the left, and 1.2° or 5.1° when displayed on the right. The starting diameter of the comparison and the assignment of the two yellow circles as comparison or standard were randomised for each participant. The down and up arrow keys decreased and increased, respectively, the diameter of the comparison circle by 0.28°. Final adjustments were recorded in pixels. These values represented the diameter that the comparison circle needed to be to perceptually match the standard circle.

### Procedures specific to the Poggendorff illusion

The illusion comprised two yellow (RGB: 255, 0, 255) transversal line-ends, angled 59.2° from the horizontal plane, on opposing sides of a contextual purple rectangle (RGB: 128, 0, 128) (Fig. 1C). Each yellow line-ends subtended 5.7°, while the contextual purple rectangle subtended 5.7° in width and 26.1° in height. This configuration typically produced the illusion of the two line-ends being displaced relative to each other, even when they were both collinear. Specifically, the line-ends on the left and right typically appeared lower and higher, respectively (Fig. 1C). Note from the figure that, unlike the other illusion tasks, we provided a template on the right side of the display as to how the standard and comparison should look like. This addition was deemed necessary to facilitate comprehension.

One of the yellow line-ends was designated as the comparison, and the other as the standard. The standard always remained in the same position, while the comparison could be moved along the vertical axis. The initial vertical position of the comparison line-end was lowered by 0.9° or raised by 4.8° when displayed on the left, and lowered by 5.6° or raised by 2.8° when displayed on the right. The down and up arrow keys moved the comparison line downwards and upwards, respectively, by 0.14°. Final adjustments were recorded in pixels as the vertical position of the comparison line relative to the top of the screen as it would cross the midline of the contextual purple rectangle. These values represented the position where the comparison line appeared to be collinear to the standard line.

### Procedures specific to the Ponzo illusion

The illusion comprised two yellow horizontal lines (RGB: 255, 0, 255) presented parallel to one another, placed over four converging purple lines (RGB: 128, 0, 128) (Fig. 1D). The latter extended from the bottom to the top of the computer screen, providing contextual cueing simulating linear perspective. The display typically produced the illusion that the yellow line on top appeared longer than the one on the right when both were the same length (Fig. 1D). One of the yellow lines was designated as the comparison, and the other as the standard. The standard always subtended 13.6°, while the comparison line was initially presented with a length of either 1.2° or 20.5°. The starting length of the comparison and the assignment of stimuli as comparison or standard were randomised for each participant. The down and up arrow keys decreased and increased, respectively, the comparison line by 0.28°. Final adjustments were recorded in pixels. These values represented the length that the comparison line needed to be to perceptually match the standard line.

### GSR recordings

To measure the GSR, two disposable electrodes connected to a Shimmer GSR3 + sensor unit (Shimmer Sensing, Dublin, Ireland) were attached to the palm of each participant’s non-dominant hand. Recordings from this sensing unit was acquired via Bluetooth using an in-house program in Matlab R2019b (MathWorks Inc., Natick, MA, USA) on a laptop PC (Latitude 7480, Dell Inc., Austin, TX, United States) running Windows 10. The sample rate was 19.5 Hz. We measured GSR continuously from the time after participants completed the handedness questionnaire until after they completed the schizotypy questionnaire. GSR detects the changes in sweat gland function by measuring skin conductance (Montagu and Coles 1966). Since stress can cause changes in sweat gland activity (Baker 2019), we inferred changes in stress levels from differences in GSR recordings. To know when events occurred in the GSR recording, the visual display computer played audio beeps. These sounds were sent to the GSR computer via a stereo 3.5 mm audio cable, which our in-house Matlab program recorded simultaneously with the GSR. Audio beeps were played when each stress condition began and ended.

### Data Preparation

Data from the illusion tasks were normalised prior to statistical analyses. Calculating a normalised index of illusion strength for each illusion allowed for more meaningful comparisons both between illusions and with results from other studies that also calculated similar indices (e.g., Chouinard et al. 2013; Schwarzkopf et al. 2011). These scores were calculated as: [(Perceived Size in Configuration A − Perceived Size in Configuration B) / (Perceived Size in Configuration A + Perceived Size in Configuration B); configuration A denoting the condition one would expect to see judgements with greater pixel values].

The GSR signal was pre-processed in Matlab in the following manner. The first nine sample points of the data were removed given the presence of a spiking artifacts when the recordings began. Then, we converted the signal from kOhms (a measure of resistance) to µS (a measure of conductance) by calculating the inverse and multiplying this value by 1,000. A high-pass filter was then applied to the signal to remove frequencies lower than 45 min, which removed linear and non-linear trends in the data. We then used the audio signal to denote the points in the GRS signal when the non-glitch and glitch conditions began and ended. Participants’ baseline stress levels were calculated as their mean GSR level throughout the entire recording, which was then used to calculate percent scores in the glitch and no-glitch conditions. Specifically, for each participant, we first subtracted the mean baseline GSR from the mean GSR for a particular condition. We then divided this value by the mean baseline GSR before multiplying that number by 100. These percent-scores were used in the statistical analyses.

### Statistical analyses

Statistical analyses were carried out using JASP 0.19.0.0 (Jefferey’s Amazing Statistics Program, University of Amsterdam, Amsterdam, The Netherlands) and GraphPad Prism 10.2.3 (GraphPad Software; Boston, MA, USA). Unless specified otherwise, all reported p values are corrected for multiple comparisons and based on two-tailed criteria.

We performed an analysis of variance (ANOVA) with Illusion (Müller-Lyer vs. Ebbinghaus vs. Poggendorff vs. Ponzo illusions*)* and Stress (No Glitch vs. Glitch) as within-subject factors on the normalised illusion strength indices. Where appropriate, Greenhouse-Geisser corrections were made for violations of sphericity, as determined by Mauchly’s test of sphericity. To examine such effects independently of schizotypal traits, we re-analysed the ANOVA analysis with the three SPQ sub-scores (Cognitive-Perceptual Deficits, Interpersonal Deficits, and Disorganisation) as covariates. Post-hoc comparison *t*-tests with Bonferroni corrections were performed to examine significant interactions and main effects. For each illusion, a one-sample *t* test against zero was performed, which reflected its strength. We report effect sizes as partial eta-squares (*η*_*p*_^*2*^) for ANOVA and *Cohen’s d* for *t*-tests.

Total SPQ scores were characterised by creating a histogram with a bin width of 5 and fitting a Gaussian curve through these values using a non-linear regression analysis. The mean, standard deviation, and range of these scores are also reported. We also tested for gender effects using an independent sample *t*-test. A difference in GSR between the no-glitch and glitch conditions was evaluated using a paired sample *t*-test.

Pearson’s *r* correlations were used to examine linear relationships between (1) SPQ scores (total and sub-factor scores) and illusion strength; and (2) GSR and illusion strength in both the non-glitch and glitch conditions. We also used the following statistical procedure to see if the presence of computer glitches created a difference in these correlation coefficients between two conditions. First, each coefficient was converted into a *z*-score (*Z*) using Fisher’s *r*-to-*z* transformation (Fisher 1915). Specifically, *Z* was calculated as: 0.5 × ln ((1 + *r*) / (1 – *r*). Then, we used the sample size obtained for each correlation coefficient to calculate final *z*-scores (*Z*_*12*_), denoting differences between two different correlation coefficients (Cohen 1996). Specifically, *Z*_*12*_ was calculated as:

$$\:{Z}_{12}=\frac{{Z}_{1}-{Z}_{2}}{\sqrt{\frac{1}{{N}_{1}-3}+\frac{1}{{N}_{2}-3}}}$$

The end-result denoted whether two correlations differed from each other.

Visual inspection of significant correlations revealed outliers in the GSR and Müller-Lyer illusion correlation in the Glitch condition. Rather than removing them from the dataset, which would create a dataset that is no longer representative of the general population, we opted to compare these results with a re-analysis using Spearman’s rank-order correlations, a non-parametric method offering more protection against outliers.

## Results

### Illusion strength

**Fig. 3:**
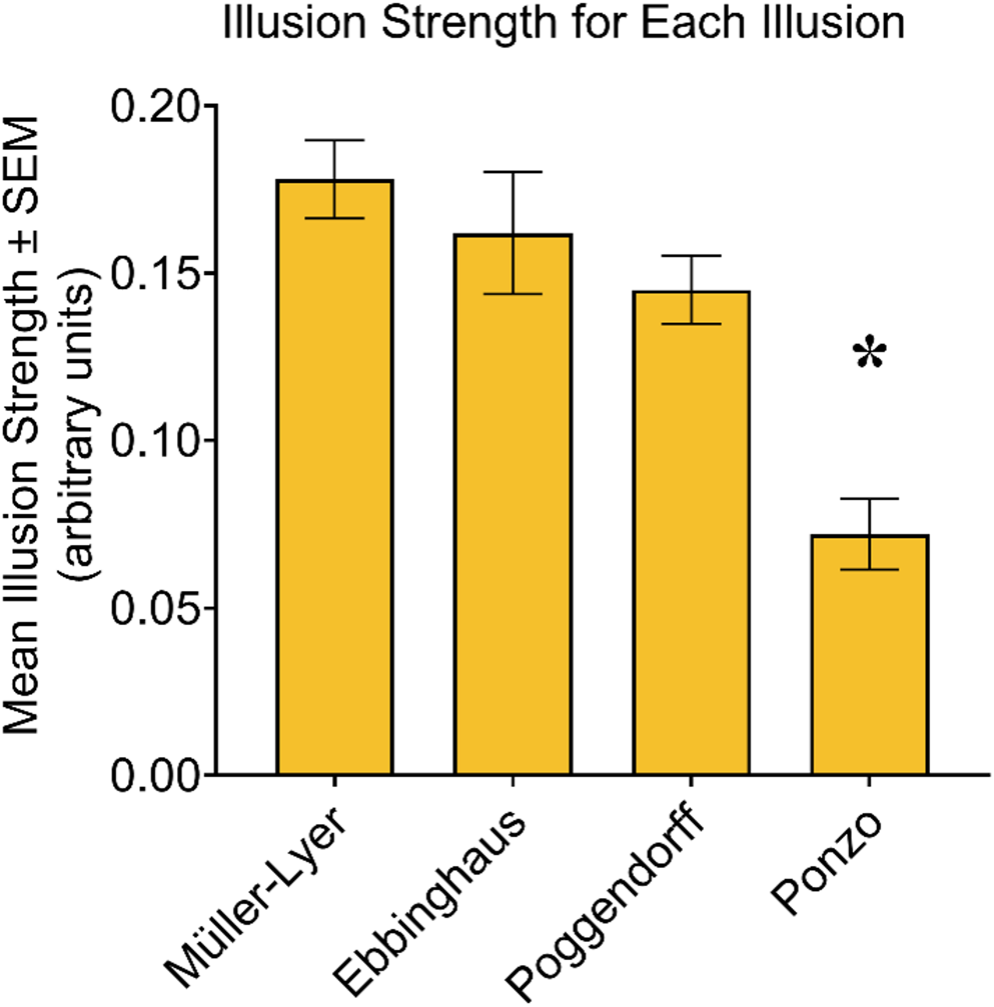
Illusion strength for each illusion. The asterisk (*) over the Ponzo illusion bar denotes a significantly weaker illusion strength compared to all the other illusions, all *p* < .001

ANOVA revealed a main effect of Illusion (*F*_*(2.4, 80.6)*_ = 13.03, *p* < .001, *η*_*p*_^*2*^ = 0.28) (Fig. 3). A main effect of Stress was not present (*F*_*(1,34)*_ = 2.69, *p* = .110, *η*_*p*_^*2*^ = 0.07) nor was there an Illusion × Stress interaction (*F*_*(2.2, 74.4)*_ = 1.15, *p* = .325, *η*_*p*_^*2*^ = 0.03). Bonferroni-corrected *t*-tests revealed a weaker susceptibility to the Ponzo illusion compared to all the other illusions (all *p* < .001). No other pairwise comparisons between illusions were significant (all *p* > .319). Illusion strength was significantly different from 0 for all illusions as determined by one-sample *t*-tests (Müller-Lyer illusion: *t*_*(34)*_ = 15.18, *p* < .001, *Cohen’s d* = 2.57; Ebbinghaus illusion: *t*_*(34)*_ = 8.85, *p* < .001, *Cohen’s d* = 1.50; Poggendorff illusion: *t*_*(34)*_ = 14.41, *p* < .001, *Cohen’s d* = 2.44; Ponzo illusion: *t*_*(34)*_ = 6.93, *p* < .001, *Cohen’s d* = 1.17). When the three sub-scores of the SPQ were entered into the ANOVA as covariates, the main effect of Illusion disappeared (*F*_*(2.3, 70.9)*_ = 2.71, *p* = .066, *η*_*p*_^*2*^ = 0.08). This re-analysis also revealed a main effect of Interpersonal Schizotypy (*F*_*(1, 31)*_ = 12.01, *p* = .002) and an Illusion × Interpersonal Schizotypy interaction (*F*_*(2.3, 70.9)*_ = 3.75, *p* = .023, *η*_*p*_^*2*^ = 0.11). As explained in the next section, the latter was driven by the presence of correlations between illusion strength and the interpersonal schizotypy subscale in some illusions but not others.

### Effects of schizotypy

**Fig. 4:**
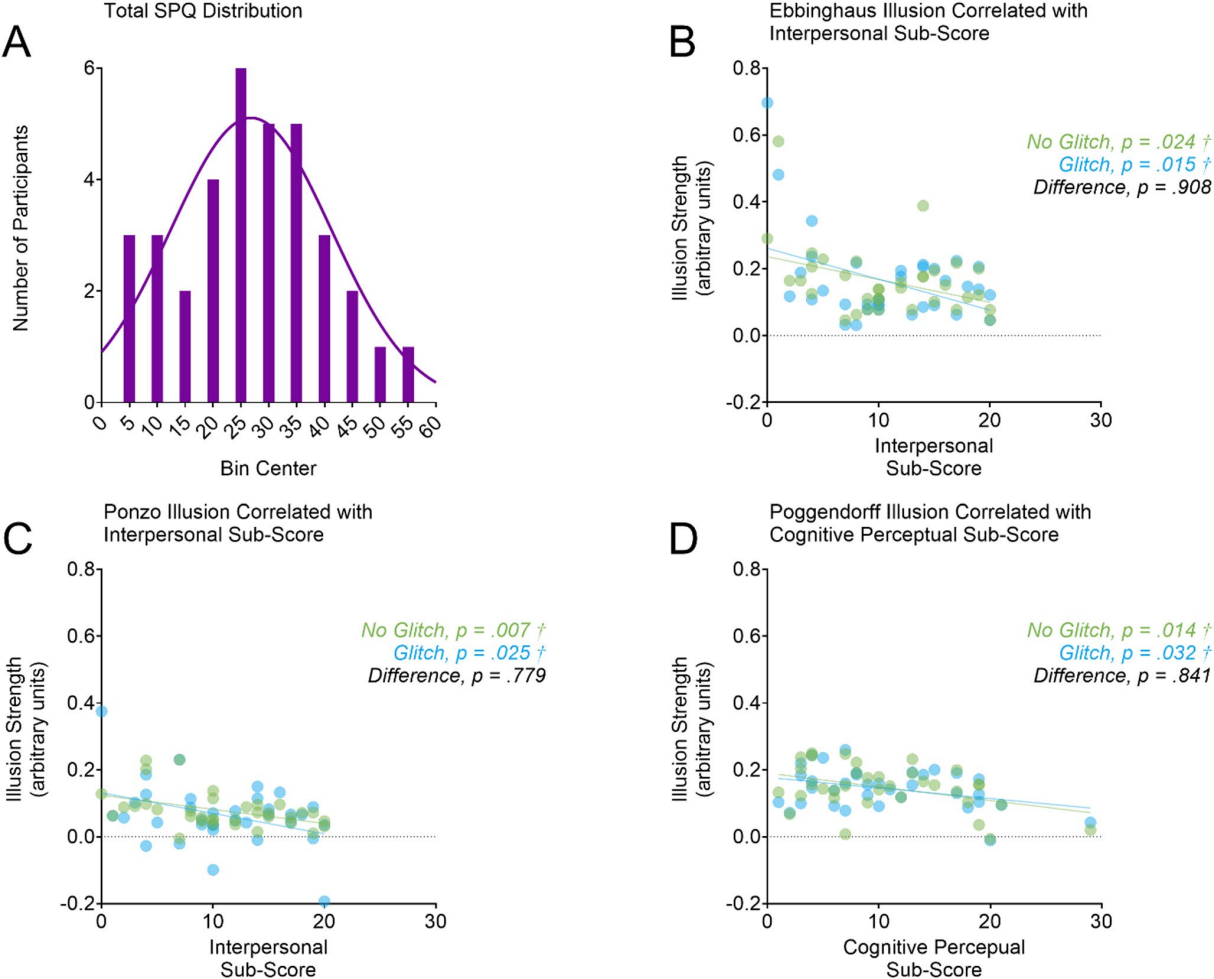
Effects of schizotypy. As shown in panel A, the total SPQ scores were normally distributed. As shown in panel B, the strength of the Ebbinghaus illusion decreased with interpersonal schizotypy in both the glitch (in blue) and no-glitch (in green) conditions. Likewise, as shown in panel C, the strength of the Ponzo illusion decreased with interpersonal schizotypy in both the glitch (in blue) and no-glitch (in green) conditions. The strength of the Poggendorff illusion decreased with cognitive-perceptual schizotypy in both the glitch (in blue) and no-glitch (in green) conditions. Daggers (†) denote significant correlations, all *p* < .05

Total SPQ scores were normally distributed with a range of 4–53 (*M* = 27.17, *SD* = 12.89, Skewness: *Z* = 0.13, Kurtosis: *Z* = −0.67) (Fig. 4A). A Gaussian curve on the histogram created from these scores fitted the data (*r*_*(11)*_ = 0.87, *p* = .012). We performed an independent samples *t* test to evaluate the effects of gender. These tests revealed that the total SPQ scores did not differ between males and females (mean difference: 5.5 points, *t*_*(33)*_ = 0.86, *p* = .260). Table 1 presents the Pearson *r* coefficients for each correlation between illusion strength and SPQ total score, and the *Z* scores for evaluating differences in correlations between the no-glitch and glitch conditions. None of the correlations between the total SPQ scores and illusion strength were significant (all *p* > .171) nor any of the Z scores for evaluating differences in correlations between the no-glitch and glitch conditions (all *p* > .412).

**Table 1:** Correlations between illusion strength and the total SPQ score

Illusion	Non-Glitch			Glitch			Difference	
	*r*_(33)_	95% CI	*p*	*r*_(33)_	95% CI	*p*	Z	*p*
Müller-Lyer	0.03	-0.30–0.36	0.854	−0.17	-0.48–0.17	0.325	0.82	0.413
Ebbinghaus	-0.24	-0.53–0.11	0.172	-0.13	-0.45–0.21	0.472	− 0.44	0.663
Poggendorff	-0.18	-0.49–0.16	0.287	-0.08	-0.40–0.26	0.643	-0.4	0.672
Ponzo	-0.20	-0.50–0.14	0.247	-0.06	-0.38–0.28	0.753	− 0.60	0.552

Significant correlations emerged when we examined how illusion strength changed with the SPQ sub-scores (Fig. 4B-D). The strength of the Ebbinghaus illusion was negatively correlated with interpersonal schizotypy in both the glitch (*r*_*(33)*_ = -0.41, 95% CI [-0.63, 0.05], *p* = .015) and non-glitch (*r*_*(33)*_ = -0.38, 95% CI [-0.65, -0.09], *p* = .024) conditions (Fig. 4B). *S*imilar findings were observed for the Ponzo illusion with higher interpersonal schizotypy being associated with reduced illusion strength in both the glitch (*r*_*(33)*_ = -0.38, 95% CI [-0.68, 0.14], *p* = .025) and non-glitch (*r*_*(33)*_ = -0.45, 95% CI [-0.63, -0.05], *p* = .007) conditions (Fig. 4C). Meanwhile, the strength of the Poggendorff illusion was negatively associated with cognitive–perceptual schizotypy in both the non-glitch (*r*_*(33)*_ = -0.41, 95% CI [-0.66, -0.09], *p* = .014) and glitch (*r*_*(33)*_ = -0.36, 95% CI [-0.62, -0.03], *p* = .032) conditions (Fig. 4D). None of the other correlations were significant (all *p* > .279). Table 2 presents the Pearson *r* coefficients for each correlation between illusion strength and the SPQ sub-scores, and the *Z* scores for evaluating differences in correlations between the no-glitch and glitch conditions. None of the Z-scores for evaluating the differences in correlations between no glitch and glitch conditions were significant (all *p* > .277).

**Table 2:** Correlations between illusion strength and the SPQ sub-scores

Illusion	Non-Glitch			Glitch			Difference	
	*r*_(33)_	95% CI	*p*	*r*_(33)_	95% CI	*p*	Z	*p*
A) Cognitive-Perceptual Schizotypy								
Müller-Lyer	0.084	-0.26–0.41	0.633	-0.169	-0.48–0.17	0.331	1.01	0.312
Ebbinghaus	-0.074	-0.40–0.27	0.671	0.044	-0.45–0.21	0.800	-0.47	0.637
Poggendorff	-0.412 †	-0.66–0.09	0.014	-0.362 †	-0.62–0.03	0.032	-0.20	0.841
Ponzo	-0.164	-0.18–0.47	0.347	0.107	-0.23–0.43	0.540	-1.08	0.278
B) Interpersonal Schizotypy								
Müller-Lyer	-0.144	-0.46–0.20	0.410	-0.181	-0.49–0.16	0.297	0.15	0.882
Ebbinghaus	-0.380 †	-0.63–0.05	0.024	-0.409 †	-0.65–0.09	0.015	0.12	0.908
Poggendorff	0.011	-0.32–0.34	0.280	0.178	-0.17–0.49	0.758	-0.67	0.504
Ponzo	-0.449 †	-0.68–0.14	0.007	-0.379 †	-0.63 – -0.05	0.025	-0.28	0.779
C) Disorganised Schizotypy								
Müller-Lyer	-0.039	-0.37–0.30	0.823	-0.157	-0.47–0.19	0.368	0.47	0.637
Ebbinghaus	-0.137	-0.45–0.20	0.432	0.097	-0.25–0.42	0.581	-0.94	0.349
Poggendorff	-0.188	-0.49–0.16	0.280	-0.054	-0.38–0.28	0.758	-0.54	0.594
Ponzo	-0.118	-0.43–0.22	0.501	0.052	-0.29–0.38	0.765	-0.68	0.497

Daggers (†) denote significant correlations, *p* < .05

### GSR and illusion strength

**Fig. 5:**
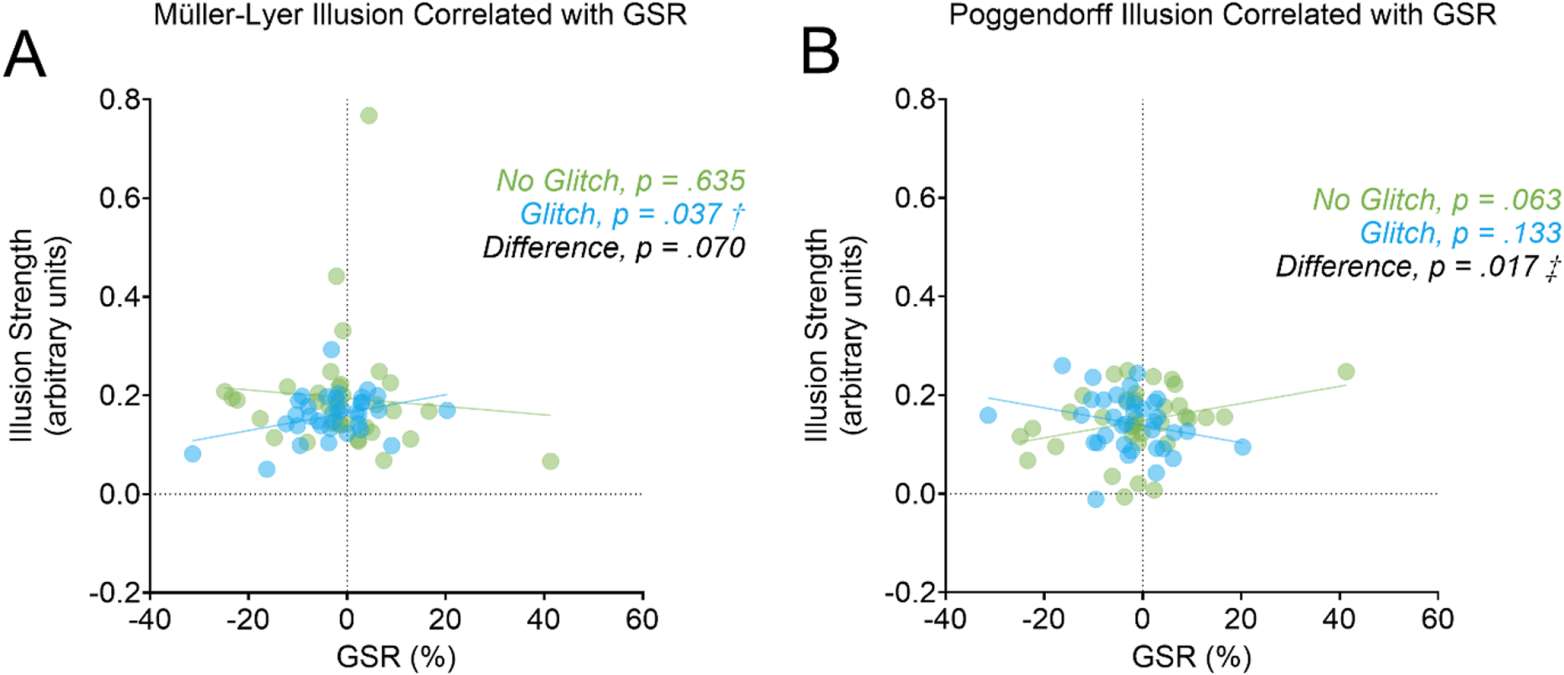
As shown in panel A, the strength of the Müller-Lyer illusion increased with GSR in the glitch (in blue) but not the no-glitch (in green) condition. There was a trend for the two correlations to differ. As shown in panel B, there was a trend for the strength of the Poggendorff illusion to increase with GSR in the no-glitch (in green) but not the glitch (in blue) condition. The dagger (†) denotes a significant correlation whilst the double dagger (‡) denotes a significant difference between correlations, both *p* < .05

A paired-sample *t*-test did not reveal differences in GSR between the no-glitch and glitch conditions (*t*_*(34)*_ = 0.64, *p* = .527, *Cohen’s d* = 0.28). Table 3 presents the Pearson *r* coefficients for each correlation between illusion strength and GSR, and the *Z* scores for evaluating differences in correlations between the no-glitch and glitch conditions. The strength of the Müller-Lyer illusion increased as a function of GSR in the glitch (*r*_*(33)*_ = 0.35, 95% CI [0.02, 0.62], *p* = .037) but not the no-glitch (*r*_*(33)*_ = −0.08, 95% CI [-0.41, 0.26], *p* = .635) condition (Fig. 5A). The difference between these two correlations approached significance (Z = −1.81, *p* = .070) (Fig. 5A). Our analyses further revealed changes in correlations between the glitch and no-glitch conditions for the Poggendorff illusion (Z = 2.38, *p* = .017) (Fig. 5B). However, this difference was the opposite of what we found for the Müller-Lyer illusion. Namely, the strength of the Poggendorff illusion tended to, but not significantly, increase with GSR in the no-glitch condition (*r*_*(33)*_ = 0.32, 95% CI [-0.02, 0.59], *p* = .062). The relationship between the strength of the Poggendorff illusion and GSR was non-significant in the glitch condition (*r*_*(33)*_ = -0.26, 95% CI [-0.55, 0.08], *p* = .133). No significant correlations or difference between correlations were found for the Ebbinghaus and Ponzo illusions (all *p* > .234). A re-analysis using Spearman’s rank correlations demonstrated a change in significance for the correlation between the Müller-Lyer illusion and GSR in the glitch condition. This correlation was no longer significant but trended towards significance (*r*_*s(33)*_ = 0.32, 95% CI [-0.01, 0.60], *p* = .058). The re-analysis of data with Spearman’s rank correlations did not alter the significance of the other results.

**Table 3:** Correlations between illusion strength and GSR

Illusion	Non-Glitch			Glitch			Difference	
	*r*_(33)_	95% CI	*p*	*r*_(33)_	95% CI	*p*	Z	*p*
Müller-Lyer	-0.08	-0.41–0.26	0.635	0.35 †	0.02–0.62	0.037	-1.81	0.070
Ebbinghaus	-005	-0.38–0.28	0.758	-0.16	-0.47–0.18	0.347	0.45	0.656
Poggendorff	0.32	-0.02–0.59	0.133	-0.26	-0.55–0.08	0.133	2.38 ‡	0.017
Ponzo	0.08	-0.26–0.40	0.654	0.21	-0.14–0.50	0.235	-0.52	0.604

The dagger (†) denotes a significant correlation whilst the double dagger (‡) denotes a significant difference between correlations, *p* < .05

## Discussion

This study aimed to examine the effects of schizotypy and acute technostress on the perception of visual illusions. The strength of the Ebbinghaus and Ponzo illusions decreased as a function of higher interpersonal schizotypy. Meanwhile, higher cognitive-perceptual schizotypy was significantly associated with a weaker Poggendorff illusion. The findings also revealed effects of acute technostress on the strength of the Müller-Lyer and Poggendorff illusions. Taken together, our results highlight that different factors of schizotypy and acute technostress can exert different influences on different visual illusions.

### Schizotypy effects on the Ponzo, Ebbinghaus, and Poggendorff illusions

Although total schizotypy had no effect on illusion strength, higher interpersonal schizotypy reduced susceptibility to the Ebbinghaus and Ponzo illusions. The question arises as to why interpersonal schizotypy would exert these effects. The interpersonal schizotypy sub-score captures constricted affect, no close friends, and heightened social anxiety. This factor is typically found in close connection with negative symptoms of schizophrenia, such as anhedonia, blunted affect, and avolition. One possible explanation for the reduced illusion strength is a shift towards a more detailed-oriented processing style. According to the Easterbrook hypothesis, heightened anxiety and negative moods can narrow attentional focus and consequently induce a more detailed-oriented processing style (Easterbrook 1959). Consistent with this account, previous studies using the Navon’s task have found a higher tendency for participants to focus on smaller stimuli, at the expense of processing a Gestalt, when both anxiety and negative mood increases (Tyler et al., 1982; Gasper 2004). Other studies using the Flanker task have found that responses to a central target are less affected by irrelevant contextual “noises” when negative mood increases (Moriya and Nittono 2011; Schmitz et al. 2009). In the present study, interpersonal schizotypal traits may have similarly biased perception toward local stimulus features, thereby weakening the influence of the contextual cues present in our Ebbinghaus (i.e., the two rings of contextual purple circles) and Ponzo (i.e., the four converging purple lines) illusion displays.

Conversely, increases in cognitive-perceptual schizotypy reduced susceptibility to the Poggendorff illusion. The illusion is thought to be driven by both low and high levels of cognitive processing based on two prominent notions to explain the illusion (Chouinard et al. 2021). The first posits that people’s perceived misalignment of the transversal line-ends arises from a tendency to overestimate acute angles and underestimate obtuse ones (Tausch 1954; Thiéry 1895). This idea is supported mechanistically from electrophysiological recording studies in the feline primary visual cortex (V1) neurons, which demonstrate that the line orientation preference for these neurons can change when a second line is presented and intersects with the first line at an angle through lateral inhibition (Burns and Pritchard 1971). Cognitive-perceptual schizotypy is unlikely to affect this mechanism directly but could more directly affect mechanisms underpinning the second prominent notion to explain the Poggendorff illusion, involving higher levels of cognitive processing associated with understanding depth through occlusion (Gillam 1971; Howe et al. 2005). Our research in the Poggendorff illusion in children demonstrate how skills associated with both language and abstract reasoning correlate more strongly with how they perceive the illusion than their performance on simpler visual discrimination tasks, underscoring the importance of high levels of cognitive processing (Chouinard et al. 2021).

The importance of high levels of cognitive processing is further demonstrated by the Poggendorff illusion still remaining present when the contextual rectangle in the display is replaced by an illusory contour of one (Day et al. 1977; Gregory 1972; Meyer and Garges 1979; Shen et al. 2016). As it turns out, individuals with psychosis exhibit deficits in visual contour integration, particularly in association with positive symptoms, such as hallucinations and delusions (Carter et al. 2017). Expanding on this idea, evidence from nonclinical samples suggest that higher cognitive-perceptual schizotypy is related to poorer local contour processing and reduced tolerance for visual noise (Panton et al. 2018). These findings suggest that elevated positive schizotypal traits may lead to a processing style with lower levels of contour integration, thereby diminishing the influence of the contextual rectangle, either present or imagined, and the strength of the Poggendorff illusion.

The observed effects of schizotypy on the strength of the Ponzo, Ebbinghaus, and Poggendorff illusions align with longstanding theories that individuals on the psychosis spectrum exhibit attenuated top-down processing (Hemsley 2005). This weakened modulation may lead to reduced use of high-level contextual cues, resulting in greater resistance to certain visual illusions (King et al. 2017). While studies in clinical populations yield mixed results, partly due to confounding factors such as medication and symptom severity (King et al. 2017), the present study focused on a nonclinical sample, allowing for a clearer examination of how distinct schizotypy dimensions relate to illusion susceptibility. These findings extend support for the top-down processing deficit hypothesis to the subclinical, healthy end of the psychosis spectrum. Furthermore, the differential associations between specific illusions and schizotypy factors highlight that visual illusions engage distinct perceptual mechanisms, and that each domain of schizotypy may exert unique influences.

It is unclear as to why the strength of the Müller-Lyer illusion did not also correlate with a sub-score of the SPQ, other than the possibility that the illusion could be underpinned by a different set of mechanisms. Indeed, in an earlier study, we performed a principal component analysis on the illusion strength of 17 illusions (Chouinard et al. 2016). The findings indicated that the Müller-Lyer, Ebbinghaus, Poggendorff, and Ponzo illusions each loaded onto separate components, suggesting that they are processed by distinct mechanisms. Further research would be required to understand how the Müller-Lyer illusion is affected in schizophrenia. It is of note that the illusion tends to increase in strength (e.g., Kantrowitz et al. 2009; Tam et al. 1998; Weckowicz and Witney 1960 Dirzius et al. 2013; Capozzoli and Marsh 1994), whilst the Ebbinghaus (e.g., Uhlhaas et al. 2006; Horton and Silverstein 2011; Tibber et al. 2013) and Ponzo (e.g., Kantrowitz et al. 2009) illusions tend to decrease in strength in this clinical population relative to control participants (for a review, see King et al. 2017). The one study that we know of that examined the Poggendorff illusion in patients with schizophrenia did not demonstrate a difference in strength compared to control participants (Kantrowitz et al. 2009).

### Technostress on the Müller-Lyer and Poggendorff illusions

A positive association was found between physiological stress responses and the strength of the Müller-Lyer illusion under the glitch condition, indicating that acute technostress, independent of schizotypy, enhanced illusion strength. While prior studies have not directly examined acute technostress in this context, our findings support the broader view that stress modulates perceptual processing, underlying the possible balance between top-down and bottom-up influences. In one of many explanations, the Müller-Lyer illusion is thought to rely on resolving visual ambiguity using prior expectations, especially depth cues (Gregory 1997). Under stress, individuals may increasingly depend on heuristics and established priors due to limited mental resources and heightened uncertainty (Yu 2016; Baror & Bar, 2020). Stress-induced mental load has been shown to impair the integration of new sensory input while enhancing reliance on existing associations (Wang et al. 2013; Herz et al. 2020). This shift can lead to perceptual rigidity, where ambiguous stimuli are resolved based on dominant prior beliefs, potentially intensifying the illusion. Within the predictive coding framework, acute technostress may bias perception toward over-weighted priors, amplifying the strength of the Müller-Lyer illusion.

The effects of technostress on the Poggendorff illusion were different and more complex. Namely, the strength of the illusion trended towards an increase with GSR in the no-glitch condition whilst there was no obvious relationship between illusion strength and GSR in the glitch condition. Although these individual correlations were not significant, we did observe a significant difference between them – which requires commenting. This significant result indicates that the effects of stress on the Poggendorff illusion is different when a stressor is present versus absent. Earlier, we proposed that the Poggendorff illusion might involve both low- and high-level mechanisms. Perhaps, it could be the case that the presence of a stressor changed the balance between these two mechanisms as levels of stress went beyond a particular threshold. Further research in a greater sample of participants would be required to provide more clarity on how stress can affect this illusion.

### Absence of stress-schizotypy joint effects

Previous studies have suggested that stress may enhance visual illusion strength in subclinical populations, such as individuals with psychotic-like experiences (Sperandio et al. 2023) and those with varying levels of schizotypy (Zouraraki et al. 2023). However, stress was conceptualized differently in those studies compared to the present one. Sperandio et al. (2023) used the Depression Anxiety Stress Scales (DASS; Lovibond and Lovibond 1995), assessing stress over a seven-day period—capturing more chronic stress. Zouraraki et al. (2023) examined stress through trauma exposure, reflecting a more severe and long-term form. In contrast, our study focused specifically on acute technostress induced during task performance. This distinction highlights a potential quantitative difference in stress types, which may account for the findings. Given that schizotypal traits and technostress can have opposing effects on visual illusion susceptibility, their subtle interaction may have cancelled each other out in our sample. It is also possible that a higher intensity or longer exposure to technostress might be required to produce perceptual changes. Thus, the joint effects of stress and schizotypy reported in previous studies may not generalize to the context of acute technostress examined here, likely due to differences in the magnitude and nature of both stress and schizotypal trait expression.

### Methodological considerations

There are four methodological considerations we wish to raise. The first relates to the absence of a significant mean difference in GSR between conditions. It is conceivable that any effects in our manipulation would have been subtle – less compared to a stressor that occurs less frequently and is more dangerous. Ethical considerations precluded us from examining a stronger stressor. Nonetheless, our procedures were sensitive enough and allowed sufficient power to measure variability in a physiological response to stress and relate this to illusion strength – as evidenced by a significant correlation in the glitch condition for the Müller-Lyer illusion. Future studies could incorporate additional physiological measures (e.g., heart rate, cortisol level) to better characterise the stress response (Mishra and Rašticová 2024; Premkumar et al. 2020). This would lead to a deeper and more nuanced understanding of how stress relates to illusion strength and how this relationship changes with schizotypy.

Second, one of the strengths of this study is the naturistic simulation of technostress. Specifically, the computer glitches were integrated with the experimental task to closely resemble a real-life scenario of acute stress when participants can encounter computer hassles and make judgements about the visual illusion. Furthermore, the implicit measure of stress response using GSR also contributed to a more objective measure on the effects of the computer glitches. It was suggested that a major pitfall in past studies of emotion and visual perception was the overuse of salient and explicit measures, which could possibly lead to demand and response biases (Firestone & Scholl, 2015). The set-up of our experiment created a naturalistic context for the stressor to be unpredictable and uncontrollable. With technostress becoming increasingly common due to rising technology use and virtual reality applications (Souchet et al. 2023), our study offers a key avenue to improve ecological validity and highlights the importance of more understanding on visual perception in everyday digital environments.

The third relates to the relatively weaker strength of the Ponzo illusion compared to the other illusions. Previous research has shown that the magnitude of the Ponzo illusion is influenced by the richness of background depth cues (Yildiz et al. 2022). For instance, the illusion appears stronger when presented in rich contextual settings, such as a railroad track background, compared to simpler configurations. In our task, the use of a minimal background may have reduced the effectiveness of the contextual cues, thereby diminishing the strength of the Ponzo illusion. More converging lines at a greater angle would have increased the strength our Ponzo illusion Fisher 1969). Nonetheless, the effects of the Ponzo illusion were sufficiently strong to allow us to find a significant correlation with interpersonal schizotypy.

Fourth, the current study had a relatively small sample that was predominantly female. Given that gender differences in technostress responses have been reported (Riedl et al. 2013), a larger sample that was more gender balanced would have allowed us to examine gender differences. Although we obtained multiple significant results, a greater sample size would provide greater confidence and would have allowed us to capture more subtle effects. For these reasons, it is recommended for future studies to replicate this study in a larger sample size with a more balanced gender composition.

## Conclusions

Our study is among the first to investigate the impact of stress and schizotypy on illusion susceptibility and the first to specifically examine the effects of technostress on visual perception. The present study has provided evidence that acute technostress and two factors of schizotypy: cognitive-perceptual and interpersonal, may be independently and uniquely associated with changes in the strength of four visual illusions: Müller-Lyer, Ebbinghaus, Müller-Lyer, Poggendorff, and Ponzo illusions. The distinctive effects of technostress and schizotypal traits on each of these illusions suggest that the processing of illusions differs and that this processing is modulated differently under the presence of acute stress and specific schizotypal traits.

## Data Availability

The data supporting the findings of this study are available on the Open Science Framework (OSF) at https://osf.io/j94wx.

## Footnotes

1 In this paper, we operationalise “low” and “high” levels of cognitive processing as reflecting mental operations that we are consciously “less” or “more” aware of, respectively.
